# The endochitinase ChiA Btt of *Bacillus thuringiensis* subsp. *tenebrionis* DSM‐2803 and its potential use to control the phytopathogen *Colletotrichum gloeosporioides*

**DOI:** 10.1002/mbo3.372

**Published:** 2016-05-12

**Authors:** Norma M. de la Fuente‐Salcido, Luz E. Casados‐Vázquez, Ada P. García‐Pérez, Uriel E. Barboza‐Pérez, Dennis K. Bideshi, Rubén Salcedo‐Hernández, Blanca E. García‐ Almendarez, José E. Barboza‐Corona

**Affiliations:** ^1^ Aniversidad Autónoma de CoahuilaEscuela de Ciencias BiológicasTorreónCoahuila27104México; ^2^ Posgrado en BiocienciasDivisión de Ciencias de la Vida, Universidad de Guanajuato Campus Irapuato‐Salamanca, IrapuatoGuanajuato36500México; ^3^ Departamento de AlimentosDivisión de Ciencias de la VidaUniversidad de Guanajuato Campus Irapuato‐SalamancaIrapuato, Guanajuato, 36500, México; ^4^ Tecnológico de Monterrey Campus QuerétaroEpigmenio González 500 FraccSan PabloQuerétaro, Qro76130México; ^5^ Department of Natural and Mathematical SciencesCalifornia Baptist University8432 Magnolia AvenueRiverside92504California; ^6^ Department of EntomologyUniversity of CaliforniaRiversideCalifornia92521; ^7^ Universidad Autónoma de Querétaro. DIPAPROPACFacultad de QuímicaQuerétaroQro, 76010México

**Keywords:** *Bacillus thuringiensis* subsp. *tenebrionis*, ChiA Btt, *Colletotrichium gloeosporioides*, DSM‐2803, endochitinase, phytopathogen

## Abstract

*Bacillus thuringiensis* subsp. *tenebrionis* DSM‐2803 has been studied extensively and spore/crystal mixtures of this strain are used widely in commercial products to control coleopteran pests. The endochitinase *chiA Btt* gene of *B. thuringiensis* subsp. *tenebrionis* DSM‐2803 was cloned and expressed in *Escherichia coli*. The recombinant 6x‐histidine tagged protein (rChiA Btt, ~74 kDa), was purified by a HiTrap Ni affinity column. The K_m_ of rChiA Btt was 0.847 *μ*mol L^−1^ and its optimal activity occurred at pH 7 and ~40°C. Most divalent cations reduced endochitinase activity but only Hg^+2^ abolished activity of the enzyme. We report for the first time the characterization of a chitinase synthesized by *B. thuringiensis* subsp. *tenebrionis* DSM‐2803, and show that the purified rChiA74 Btt reduced the radial growth and increased the hyphal density of *Colletotrichium gloeosporioides,* the etiological agent of “anthracnose” in plants.

## Introduction

Chitin, a *β*‐1‐4‐linked polymer of *N*‐acetylglucosamine (GlcNAc), is the second most abundant structural polysaccharide present in nature, eclipsed only by cellulose. Chitin is characteristically found in the exoskeleton of arthropods (insects, crustaceans) and in the cell wall of fungi and algae. Bacteria do not synthesize chitin, but many species secrete chitinolytic enzymes that hydrolyze the polymer to release units of GlcNAc for use as carbon or nitrogen sources (Bhattacharya et al. 2007). *Bacillus thuringiensis* is a well‐known bacterium that is widely used as an insect larvicide owing to the toxic effect of its proteinaceous Cry (crystal) and Cyt (cytolytic) toxins. *B. thuringiensis* strains are toxic to larvae of certain members of Lepidoptera, Diptera, and Coleoptera, such as, respectively, *B. thuringiensis* subsp. *kurstaki* HD‐1*, B. thuringiensis* subsp. *israelensis* and *B. thuringiensis* subsp. *tenebrionis* (Park et al. 1998; Barboza‐Corona et al. 2008). In comparison to Cry and Cyt toxins, chitinases of *B. thuringiensis* have been less studied, but more recently have gained attention mainly because these enzymes have potential applied value to control phytopathogenic fungi (Driss et al. 2005; Morales de la Vega et al. 2006).

Most studies on the inhibitory effect of *B. thuringiensis* to phytopathogenic fungi have been performed using bacterial suspensions, supernatants or concentrated crude preparations that contain chitinases, proteases, and other metabolites (Ramírez‐Reyes et al. 2004; Driss et al. 2005; Choi et al. 2007; Asril et al. 2014; Martínez‐Absalón et al. 2014). Only one of these studies reported the inhibitory effect of purified chitinase from *B. thuringiensis* subsp. *aizawai* against different phytopathogenic fungi (Morales de la Vega et al. 2006). However, *Colletotrichum gloeosporioides* was not included in that study and the sequence of the chitinase was not reported. *C. gloeosporioides* is a phytopathogenic fungus that causes “anthracnose” in plants, and is responsible for significant economic loss of fruit production worldwide. For example, in México, this fungus is a serious problem in reducing avocado (*Persea americana*) yield (Rodríguez‐López et al. 2013). To our knowledge, only two studies have been reported on the use of metabolites produced by *B. thuringiensis* to control this fungus, that is, partial purified chitinase from *B. thuringiensis* SAHA 12.08 (Asril et al. 2014) and a lipopeptide from *B. thuringiensis* CMB26 (Kim et al. 2004).

Chitinase gene sequences from different strains of *B. thuringiensis* have been deposited in the GenBank database (www.ncbi.nlm.nih.gov/genbank/), but only ~30% of the encoded chitinases have been characterized (Thamthiankul et al. 2001; Barboza‐Corona et al. 2003; Casados‐Vázquez et al. 2015; Ni et al. 2015). In particular, *B. thuringiensis* subsp. *tenebrionis* DSM‐2803 has been studied extensively and spore/crystal mixtures of this strain are used widely in commercial products (e.g. Novodor, Trident) to control coleopteran pests (Park et al. 1998). However, to the best of our knowledge there is no report on the cloning and characterization of a chitinase synthesized by this commercial bacterium, nor are there reports on the use of purified chitinases from *B. thuringiensis* exerting inhibitory effect against *C. gloeosporioides*. To increase our knowledge of the diversity of chitinases produced by *Bacillus* species, and for applied purposes, we cloned, sequenced, and characterized an endochitinase (ChiA Btt) of *B. thuringiensis* subsp. *tenebrionis* DSM‐2803 and demonstrated the inhibitory effect of the purified enzyme against *C. gloeosporioides*.

## Materials and Methods

### Bacterial strains and plasmids

*B. thuringiensis* subsp. *kurstaki* HD1 (Bt HD1), *B. thuringiensis* subsp. *kurstaki* HD73 (Bt HD‐73), and *B. thuringiensis* subsp. *tenebrionis* (Btt) DSM‐2803 are sporogenic bacteria kindly provided by Jorge Ibarra (CINVESTAV Irapuato, Mexico). The chitinase gene (*chiA Btt*) was cloned from *B. thuringiensis* subsp. *tenebrionis* DSM‐2803, a bacterium that is toxic to coleopteran larvae owing to its quadrangular‐flat crystal composed of Cry3 protein protoxins (~74 kDa). Recombinant plasmids were propagated in *Escherichia coli* TOP10 (Invitrogen, Carlsbad, CA) and *E*. *coli* BL21 Rosetta 2 (Merck Millipore, MA) for the purpose of cloning the gene, and for production and purification of the enzyme, respectively. Plasmids pCR 4‐TOPO (Invitrogen), pCold I (Takara Bio Inc, Otsu, Shiga, Japan), or pHT3101 (Barboza‐Corona et al. 2009) were used as cloning vectors. The first two plasmids are able to replicate in *E. coli*, whereas the pHT3101 is a shuttle vector with two replication origins, one for *E. coli* and the other for *B. thuringiensis*.

### Chitinase activity of *B. thuringiensis* strains

To test the chitinolytic activity of *B. thuringiensis* strains, ~1 × 10^8^ cells mL^−1^ were used to inoculate nutrient broth (DB Bioxon), and cultures were grown at 28°C, 180 rpm for ~72 h to reach autolysis. Samples were collected in duplicate to determine the optical density at 600 nm, using a Smart Spec3000 (BioRad, Hercules, CA). Three fluorogenic chitin derivatives, 4‐methylumbelliferyl‐*β*‐D‐*N,N′,N′′‐*triacetylchitotriose [4‐MU‐(GlcNAc)_3_] (tetrameric fluorescent derivative), 4‐methylumbelliferyl‐*β*‐D‐*N,N′‐*diacetylchitobioside [4‐MU‐(GlcNAc)_2_] (trimeric fluorescent derivative), and 4‐methylumbelliferyl‐N‐acetyl‐*β*‐D‐glucosaminide [4‐MU‐GlcNAc] (dimeric fluorescent derivative) (Sigma, St. Louis, MO) were used to evaluate endochitinase, chitobiosidase, and N‐acetylglucosaminidase activities, respectively, in a 100 mmol L^−1^ phosphate reaction buffer (pH 7.0) (Barboza‐Corona et al. 2003). 4‐MU released from the fluorogenic substrate was calculated fluorometrically with excitation at 360 nm and emission at 455 nm (Glomax Multi Jr. Detection System, Promega, Sunnyvale, CA), using a 4‐MU standard curve. One unit (U) of chitinolytic activity was defined as the amount of enzyme required to release 1 *μ*mol of 4‐methylumbelliferone in 1 h (Barboza‐Corona et al. 2003).

### Cloning of *chiA* Btt, *rchiA Btt* and nucleotide sequence of the gene

The chitinase gene of Btt (*chiA* Btt) was amplified by the polymerase chain reaction (PCR), using primers based on conserved regions in *chiA74*. Oligonucleotides chiA74–1 (5‐ACGCGTCGACCTTTCTACGTCTTTAATAATTGGCTCCATAC‐3) and chiA74‐3 (5‐AACTGCAGCGAAAGCCTTTCCCTAACAGGTGACTATC‐3) allowed the amplification of *chiA* Btt from its native promoter to the putative transcriptional terminator. Gene amplification was performed using genomic DNA, as described previously (Barboza‐Corona et al. 2009), with an initial denaturation at 94°C for 2 min, followed by 35 cycles of denaturation at 94°C for 30 sec, annealing at 56°C for 1 min, an extension at 72°C for 2 min and final extension of 72°C for 10 min. The amplicon was cloned into the pCR4‐TOPO (Invitrogen) and *E. coli* TOP10 was transformed (Invitrogen) to obtain the recombinant strain *E. coli* TOP10/*chiA* Btt‐ pCR4‐TOPO. The recombinant plasmid, *chiA* Btt‐ pCR4‐TOPO, was used to determine the nucleotide sequence of *chiA* Btt by dideoxynucleotide sequencing using the chiA74‐1 and chiA74‐3 primers, as well as internal primers to obtain overlapping sequences using a 3730 XL DNA analyzer (Applied Biosystems). For comparative analyses, the Blast programs in the NCBI database (http://www.ncbi.nlm.nih.gov) and DNAStar Version 5 (DNASTAR, Inc. Madison, WI) were used. The sequence of the *chiA* Btt gene was submitted to the GenBank nucleotide database (www.ncbi.nlm.nih.gov) under the accession number KJ764712.

The endochitinase *chiAΔsp Btt* gene lacking its secretion signal peptide sequence was amplified from *chiA* Btt‐pCR4‐TOPO with chiA74‐34‐Nter/HindIII (5′CCGCCGAAGCTTGATTCACCAAAGCAAAGTCAAAAA3′) and chiA74‐Cter/*Hind*III (5′GCCGCCAAGCTT *CTA*GTTTTCGCTAATGACGGCATT 3′), forward and reverse primers, respectively (Casados‐Vázquez et al. 2015). Amplification was carried out as described above for *chiA* Btt. The amplicon (~2 kbp) and cold shock expression vector (pCold I) (Takara Bio Inc, Otsu, Shiga, Japan) were digested with *Hind*III and then purified using the gel extraction kit (Qiagen, Valencia, CA, USA). The vector was dephosphorylated and the amplicon was ligated into pCold I overnight at 16°C. *E. coli* TOP10 was transformed into the recombinant plasmid (pCold I‐*chiA*Δ*sp* Btt) and selected with ampicillin (100 *μ*g mL^−1^). For endochitinase expression and purification of the recombinant 6x‐histidine‐tagged protein (hereafter rChiA Btt), the calcium competent *E. coli* BL21‐Rosetta 2 was transformed into pCold I‐*chiA*Δ*sp* Btt. The recombinant protein rChiA Btt contained the *N*‐terminal end coded by ChiAΔsp Btt fused to a heterologous sequence of 28 amino acid including 6x histidine tag and Factor Xa cleavage sites coded by pCold I (Casados‐Vázquez et al. 2015).

### Determination of the chitinase activity, purification and zymogram

*E. coli* TOP10/*chiA* Btt‐ pCR4‐TOPO was grown overnight in Luria‐Bertani (LB) broth and 1 mL of culture was collected and centrifuged. Cell‐free supernatants were used to determine the chitinase activity at pH 6.8 using 4‐MU‐(GlcNAc)_3_, 4‐MU‐(GlcNAc), and 4‐MU‐GlcNAc, as previously described (Barboza‐Corona et al. 2003).

To purify the endochitinase ChiAΔsp Btt, recombinant *E*. *coli* BL21 Rosetta2*/*pCold I‐ *chiA*Δ*sp* Btt was grown overnight in 2.5 mL of LB broth supplemented with ampicillin (100 *μ*g mL^−1^) and chloramphenicol (34 *μ*g mL^−1^), and then transferred into a 250 mL fresh medium supplemented with the same antibiotics. The culture was grown at 37°C and 200 rpm to reach an OD_600_ of 0.4–0.6, and immediately incubated at 15°C for 30 min. At this point, IPTG (Isopropyl‐beta‐D‐thiogalactopyranoside) was added to a final concentration of 0.5 mmol L^−1^, and the culture was incubated at 16°C for 24 h at 200 rpm. The culture was centrifuged and the supernatant was discarded. The pellet was resuspended in 20 mL of buffer A (100 mmol L^−1^ Tris‐HCl pH 7, 150 mmol L^−1^ NaCl, 10 mmol L^−1^ imidazole) and then sonicated ten times, 30 sec each, at an amplitude of 30 Hz, using a 20 kHz ultrasonic processor (Sonic and Materials, Inc., Newtown, CT). The extract was centrifuged 30 min at 13000 *g* and the supernatant was passed through an HiTrap Ni affinity column (GE Healthcare Bio‐Sciences AB, Upsala Sweden) pre‐equilibrated with buffer A. Unbound protein was removed with 10 mL of buffer A, 10 mL of buffer A‐20 mmol L^−1^ imidazole, 10 mL of buffer A‐40 mmol L^−1^ imidazole, and rChiA74 was finally eluted with 2 mL of buffer A‐400 mmol L^−1^ imidazole. Dialysis was performed in buffer A without imidazole and protein concentration was determined using the Quick Start Bradford 1× Dye reagent (BioRad). Samples were loaded onto a Superdex 200 10/300 GL (GE Healthcare Life Science) column previously equilibrated with buffer A (100 mmol L^−1^ Tris–HCl pH 7.0, 150 mmol L^−1^ NaCl), and rChiA Btt was separated using a size‐exclusion column by fast protein liquid chromatography (FPLC) (Biologic Duo‐Flow Pathfinder 20 System BioRad, Hercules, CA). Fractions of 1 mL were collected at a rate of 0.5 mL min^−1^ using buffer A and monitored at 280 nm. In order to determine the molecular mass of rChiA Btt, fractions of purified sample were treated with Laemmli's disruption buffer supplemented with *β*‐mercaptoethanol and analyzed by sodium dodecyl sulfate (SDS)‐polyacrylamide gel electrophoresis (SDS‐PAGE). After separation by SDS‐PAGE, proteins were renatured by removing SDS and *β*‐mercaptoethanol with casein‐EDTA wash buffer [1% (w/v) casein, 2 mmol L^−1^ EDTA, 40 mmol L^−1^ Tris‐HCl (pH 9)]. Detection of chitinase activity after gel electrophoresis was performed, using the 4‐MU‐GlcNAc_3_ (Barboza‐Corona et al. 2003, 2009). Additionally, endochitinase activity was determined in triplicate assays at 37°C in 100 mmol L^−1^ phosphate buffer, pH 7, using the substrate 4‐methylumbelliferyl *β*‐D‐N,N′,N′′‐triacetylchitotrioside (4‐MU‐GlcNAc_3_) (Sigma) and purified rChiA74 at final concentrations of, respectively 2.5 *μ*mol L^−1^ and 0.8 nmol L^−1^, similar as previously described (Casados‐Vázquez et al. 2015).

### Effect of pH and temperature

The enzymatic activity of concentrated mature rChiA Btt was evaluated at 37°C with the 4‐MU‐GlcNAc_3_ at a pH range of 4–10 using a reaction buffer containing acetic acid, MES [2(N‐morpholino) ethane sulfonic acid], H_3_PO_4_, Trizma base [Tris(hydroxymethyl) aminomethane] and glycine, with a final concentration of 15 mmol L^−1^ for each component. Also, chitinase activity within a range of temperatures (5–80°C) was measured at pH 7.0 These assays were repeated three separate times in triplicate.

### Effect of divalent ions

Purified rChiA Btt (60 nmol L^−1^) was incubated with 1, 5, and 10 mmol L^−1^ of salt solutions (BaCl_2_, SnCl_2_, HgCl_2_, CaCl_2_, MnCl_2_, MgCl_2_, CuCl_2_, and ZnCl_2_) for 10 min at room temperature in 100 mmol L^−1^ acetate buffer pH 5. Seven microliters of the treated enzyme was used to perform assays in triplicate at 37°C, using the substrate 4‐MU‐GlcNAc_3_ in 100 mmol L^−1^ phosphate buffer at pH 7 as explained above.

### Determination of kinetic constants

Kinetics experiments were performed using purified rChiA Btt and the 4‐MU‐GlcNAc_3_ substrate. Reaction mixtures were incubated at 37°C in 100 mmol L^−1^ phosphate buffer (pH 7.0), using 0.8 nmol L^−1^ purified chitinase and 0.25, 0.5, 0.75, 1, 1.25, 2, 4, 8 *μ*mol L^−1^ of 4‐MU‐GlcNAc_3_. The amount of 4‐MU released from the fluorogenic substrate was calculated spectrofluorometrically from a calibration curve, using the same concentration of substrate with an excess of enzyme to convert all the substrate to product. The released fluorogenic product was measured fluorometrically, as described above. Assays were repeated in triplicate. Initial velocities were used to calculate kinetic constants (V_max_, K_m_), using GraphPad Prism Version 6.04 (www.graphpad.com) (Casados‐Vázquez et al. 2015).

### Antifungal activity of rChiA Btt against *C. gloeosporioides*

The effect of rChiA Btt on fungal growth was determined by the well diffusion method and by inhibition of fungal growth on agar plate, using rChiA Btt incorporated into the medium. In the diffusion method, wells, 8 mm in diameter, were dug into fresh potato dextrose agar (PDA) and stored for 2 h at 37°C. One disk of 8 mm of the outer edge of a 5–7‐day culture of *C. gloeosporioides* was cut with a sterile cork borer and placed into the center of fresh PDA plate. Then different quantities (0, 1.17, 3.34, 4.69, 9.38, 18.75 U) of rChiA Btt were added to each well and incubated overnight at 4°C to allow diffusion of the enzyme. Plates were incubated at 28°C for 5–7 days after which growth inhibition or morphological changes were recorded. Each assay was repeated in triplicate. To assay inhibition of fungal growth in medium incorporated into rChiA Btt, 7‐day culture of *C. gloeosporioides* was inoculated in the center PDA mixed with different rChiA Btt concentrations (0, 0.23, 0.47, 0.94, 1.88, 3.75 U mL^−1^ culture medium) and incubated at 28°C for 5–7 days. Radial growth was recorded each 24 h in millimeters (mm). In each assay, cultures without chitinase were used as controls. All assays were performed in triplicate.

## Results

### Chitinase activity of *B. thuringiensis* subsp. *tenebrionis* DSM‐2803 compared with other *B. thuringiensis* strains

**Figure 1:**
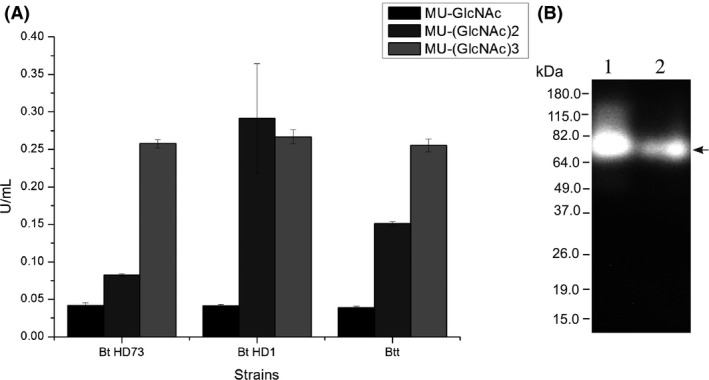
Chitinolytic activity of *B. thuringiensis* subsp. *tenebrionis* DSM‐2803 compared with other strains. (A) Determination of the chitinolytic activity of *B. thuringiensis* subsp. *kurstaki* HD73 (Bt H73), *B. thuringiensis* subsp. *kurstaki* HD1 (Bt HD1) and *B. thuringiensis* subsp. *tenebrionis* DSM‐2803 (Btt) using secreted proteins collected at 36 h. Black, dark gray, and gray columns indicated that activity was determined with MU‐GlcNAc, MU‐(GlcNAc)_2_, and MU‐(GlcNAc)_3_, respectively. (B) Zymogram using secreted proteins from both *B. thuringiensis* subsp. *tenebrionis* DSM‐2803 (lane 1) and recombinant *E. coli* TOP10/*chiA* Btt‐ pCR4‐TOPO (lane 2). Activity was detected using MU‐(GlcNAc)_3_. Arrow indicates the position of endochitinase ChiA Btt.

When the chitinolytic activity of secreted protein preparations of *B. thuringiensis* subsp. *tenebrionis* (Btt) was assayed using fluorogenic chitin compounds, the highest hydrolytic activity was obtained with 4‐MU‐(GlcNAc)_3_ (~250 mU mL^−1^), followed by 4‐MU‐(GlcNAc)_2_ (~150 mU mL^−1^) and 4‐MU‐GlcNAc (~30 mU mL^−1^). This indicated that the main chitinolytic activity of *B. thuringiensis* subsp. *tenebrionis* DSM‐2803 is an endochitinase, which is comparable to those of *B. thuringiensis* subsp. *kurstaki* HD73 and *B. thuringiensis* subsp. *kurstaki* HD1, as they showed activities of ~250 mU mL^−1^, 250 mU mL^−1^ and 270 mU mL^−1^, respectively (Fig. 1A), similar to the activity previously reported for *B. thuringiensis* subsp. *kurstaki* HD73 (Barboza‐Corona et al. 2009).

### Cloning and sequence analysis of ChiA Btt and comparison with other chitinases and chitin‐binding proteins

When secreted proteins of *B. thuringiensis* subsp. *tenebrionis* DSM‐2803 were resolved by SDS‐PAGE and analyzed by zymogram, using 4‐MU‐(GlcNAc)_3_ as a substrate, an endochitinase of ~74 kDa was detected (Fig. 1B). Subsequently, we cloned a chitinase gene from Btt (i.e. *chiA* Btt), including its native signal peptide, in *E. coli* TOP10 using the pCR4‐TOPO vector, and we recovered the recombinant chitinase from culture supernatant. The higher activity of ChiA Btt was obtained with 4‐MU‐(GlcNAc)_3_ followed by 4‐MU‐(GlcNAc)_2_ and 4‐MU‐GlcNAc (data not shown). Analyses by SDS‐PAGE and zymograms of secreted proteins confirmed that ChiA Btt was an endochitinase with the predicted molecular mass of ~74 kDa (Fig. 1B).

Sequence analyses of the *chiA* Btt gene showed that it harbored an open reading frame (ORF) that coded for a putative protein composed of 676 amino acids with a deduced molecular mass of 74.2 kDa and a predicted isolectric point of 5.74 (EditSeq, DNASTAR). The SignalP 4.1 Server predicted a signal peptide of 3.8 kDa with a cleavage site between Ala‐34 and Asp‐35 (Petersen et al. 2011). When ChiA Btt amino acid sequence was compared with those reported for other chitinases of *B. thuringiensis*, the lowest identity (78%) was observed with a chitinase of *B. thuringiensis* subsp. *pakistani* (accession number U89796) (Thamthiankul et al. 2001). However, most of the chitinases of *B. thuringiensis* showed high identity with values from 93% to 99% when compared with ChiA Btt.

**Figure 2:**
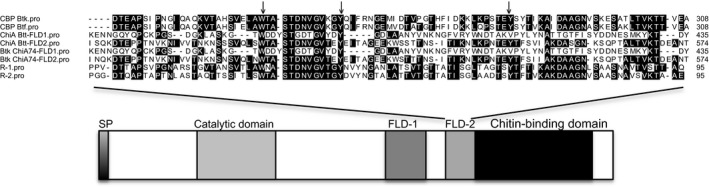
Comparison between the fibronectin‐like domain (fibronectin‐like domains, [FLD]) among chitin binding proteins, and schematic representation of the ChiA Btt modular architecture. Aromatic amino acids are indicated by arrows. Sequences are from *B. thuringiensis* subsp. *kurstaki* (chitin‐binding proteins [CBP Btk, ACW83015.1), *B. thuringiensis* (CBP Btf, WP_000795730.1), *B. thuringiensis* subsp. *tenebrionis* DSM‐2803 (ChiA Btt, GenBank accession number KJ764712), *B. thuringiensis* subsp. *kenyae* (ChiA74Btk, AF424979), and *B. circulans* ChiA (R1, R2, see Li and Greene 2010).

ChiA Btt has a modular architecture: a catalytic domain in the *N*‐terminal region belonging to the family 18 of glycosyl hydrolases, followed by two fibronectin‐like domains (FLD) and a chitin‐binding domain (CBD) in the *C*‐terminal region, which has characteristic aromatic groups of the chitin‐binding domain type 3 (http://smart.embl-heidelberg.de/smart/do_annotation.pl?DOMAIN=ChtBD3) (Fig. 2). The catalytic domain (Gly‐147 through Ser‐222) has the conserved motif “D‐X‐D‐X‐E” (i.e. Asp‐207, Asp‐209, and Glu‐211) (Li and Greene 2010). This conserved motif has been found in different orthologues of bacterial chitinases, such as *B. circulans* (accession number P20533), *Clostridium paraputrificum* (AB012764), *Enterobacter aglomerans* (U59304), and *Serratia marcescens* (B015996), among others. The critical role in catalysis by aspartic and glutamic acid residues in the conserved motif has been demonstrated by mutational approaches in different bacterial chitinases, including those of *B. circulans, S. marcescens*, and from the hyperthermophilic archaeon, *Pyrococcus furiosus* (Watanabe et al. 1993; Tsuji et al. 2010).

Following the catalytic domain are two fibronectin‐like domains: ChiA Btt‐FLD1 (Lys‐350 through Tyr‐435) and ChiA Btt‐FLD2 (Ile‐479 through Thr‐574). ChiA Btt‐FLD1 and ChiABtt‐FLD2 showed low identities to FLDs R‐1 and R‐2 of *B. circulans* (14 and 36%, respectively), but are similar to the FLDs of ChiA74 of *B. thuringiensis* subsp. *kenyae* with identities of 100% and 94%, respectively. ChiABtt‐FLD2 has ~38% identity with chitin‐binding proteins (CBP) of *B. thuringiensis* (ACW83015, WP‐000795730), and conserved aromatic amino acids Trp‐507, Tyr‐519, and Tyr‐546 (Fig. 2) (Arora et al. 2013). Finally, the *C*‐terminus of ChiA Btt, the CBD (Val‐587 through Lys‐629) shows a high identity with chitinases of *B. thuringiensis* subsp. *kenyae* ChiA74 (Barboza‐Corona et al. 2003) and ChiA HD73 of *B*. subsp. *thuringiensis kurstaki* (Barboza‐Corona et al. 2008) with values of 100% and 98%, respectively. Indeed, the highly conserved aromatic residues (Trp‐591, Tyr‐595, and Trp‐626) in the CBDs are also present in the chitin‐binding domain of ChiA Btt (Ferrandon et al. 2003; Hardt and Laine 2004).

### Purification and kinetic constant values of recombinant Btt endochitinase lacking its secretion peptide

**Figure 3:**
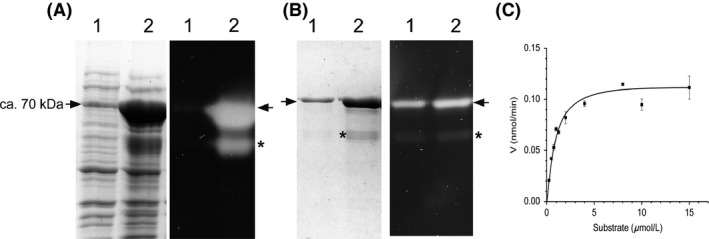
Synthesis of recombinant endochitinase in *E*. *coli* BL21 Rosetta 2 and purification of 6xHis tagged‐ChiA BttΔsp (rChiA Btt). (A) sodium dodecyl sulfate (SDS)‐PAGE (left) and zymogram (right) using 4MU‐(GlcNAc)_3_ (Sigma) as the substrate; noninduced cells (lane 1) and IPTG induced cells (lane 2). (B) Separation of rChiA Btt by HiTrap Ni affinity column. SDS‐PAGE (left), Zymogram (right); sample from second wash with buffer A‐20 mmol L^−1^ imidazole (lane 1); eluted protein with buffer A‐500 mmol L^−1^ imidazole (lane 2). Arrow indicates the position of a protein of ca. 74 kDa corresponding to rChiA Btt and the asterisk shows the location of a protein of ~55 Da that was co‐purified. (C) Determination of the Km of purified rChiA Btt using the dependence of initial velocity of substrate concentration.

The sequence encoding the recombinant endochitinase (rChiA Btt) that lacked its secretion signal peptide and contained a 6x‐His tag was expressed in *E*. *coli* BL21 Rosetta 2 using the pCold I expression vector. The recombinant *E. coli* (*E*. *coli* BL21 Rosetta2*/*pCold I‐*chiA*Δ*sp* Btt) was grown under induced conditions and rChiA Btt was obtained after cell disruption. The enzymatic activity of the intracellular rChiA Btt obtained after cell disruption was assayed with three fluorescent substrates. The highest activity (73.50 ± 3.2 U mg^−1^ protein) was observed with 4‐MU‐(GlcNAc)_3_, followed by 4‐MU‐(GlcNAc)_2_ (6.50 ± 0.50 U mg^−1^ protein) and 4‐MU‐GlcNAc (0.20 ± 0.00 U mg^−1^ protein). We did not observe activity with control *E*. *coli* BL21 Rosetta 2. This assay confirmed that the main activity of rChiA Btt is as an endochitinase. When samples were analyzed by SDS‐PAGE, the concentration of rChiA Btt in the IPTG‐induced cultures was higher than that obtained from noninduced cultures, which is in agreement with the signal intensity observed in the zymograms. As expected, SDS‐PAGE and zymogram analyses confirmed the molecular mass of mature ChiA Btt (~74 kDa). When samples from IPTG‐induced cell cultures were purified on a Ni affinity column, a protein of ~55 kDa co‐purified with the ~74 kDa protein (Fig. 3A–B) (Casados‐Vázquez et al. 2015). During UV exposure to detect fluorescence, we did not observe a signal in the noninduced sample (Fig. 3A, lane 1) as the expression was too low as compared with the induced sample. Following Ni affinity column chromatography, we were able to obtain a yield and purification of 40.27% and 4.39‐fold, respectively. Subsequently, when samples that were purified by Ni affinity were subjected to size‐exclusion chromatography, we obtained a yield of 19.72% and a purification of eightfold.

Using 4‐MU‐(GlcNAc)_3_ as the substrate, the Vmax and Km of recombinant purified rChiA Btt were 0.116 nmol min^−1^ (±0.005) and 0.847 *μ*mol L^−1^ (±0.078), respectively (Fig. 3C).

### Effect of pH, temperature and divalent cations on rChiA Btt activity

**Figure 4:**
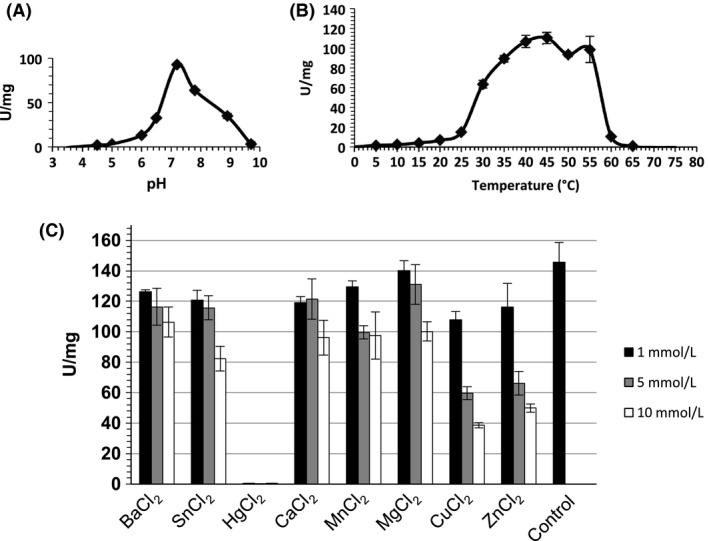
Effect of pH (A), temperature (B) and divalent cations (C) on the enzymatic activity of rChiA Btt, as determined flurometrically following hydrolysis of 4‐Mu‐(GlcNAc)_3_. In (C), the control was made by a mix reaction not supplemented with cations. Standard deviations are shown (vertical lines).

The activity of rChiA Btt was assayed in a gradient of pHs and temperatures with the 4‐MU‐(GlcNAc)_3_. The maximun enzymatic activity of rChiA Btt was observed at pH 7 and 40–45°C (Fig. 4A–B). Furthermore, all cations (1 mmolL^−1^, 5 mmol L^−1^, 10 mmol L^−1^) used in the assay reduced the enzymatic activity of rChiA Btt, and only Hg^+2^ abolished the rChiA Btt activity at the three different concentrations used (Fig. 4C).

### Antifungal activity of rChiA Btt

**Figure 5:**
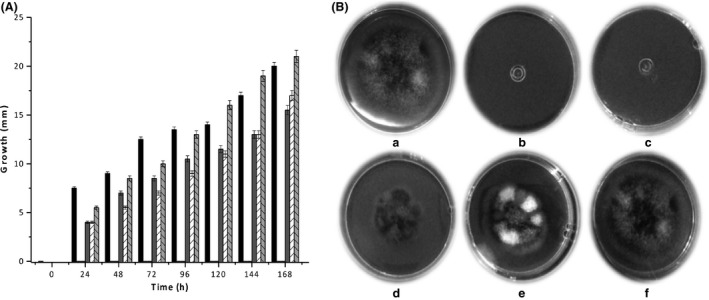
Inhibitory effect of rChiA Btt on *Colletotrichium gloeporoides*. Effect on the hyphal radial growth using potato dextrose agar supplemented with the purified chitinase (A). The inhibitory effect was evaluated during 7 days; radial growth was recorded each 24 h; medium without chitinase (black rectangle); gray rectangle, medium with 0.94 U mL^−1^ (gray rectangle), medium with 0.47 U mL^−1^ (white rectangle with lines), medium with 0.23 U mL^−1^ (gray rectangle with lines). No radial growth was observed when medium was mixed with 1.88 and 3.75 U mL^−1^. (B) Observation of radial growth in petri dish at 7 days. (a) Medium without chitinase or supplemented with (b) 3.75 U mL^−1^, (c) 1.88 U mL^−1^, (d) 0.94 U mL^−1^, (e) 0.47 U mL^−1^, (f) 0.23 U mL^−1^.

At 7 days, using 3.75 U mL^−1^ and 1.88 U/mL of rChiA Btt, no detectable growth of *C. gloeosporioides* was observed, whereas using 0.94 and 0.47 U mL^−1^, radial growth was reduced by ~25% and ~12.5%, respectively. We did not observe any notable effects in radial growth of the fungus, using 0.23 U mL^−1^ (Fig. 5).

The effect of the endochitinase against *C. gloeosporioides* was also assayed using the well‐diffusion method. In this assay, the higher chitinase concentration (18.75 U) inhibited growth of the fungus, and no effect was observed using 1.17 U of rChiA Btt. We also note that, based on microscopic examination, the higher chitinase concentration, the higher hyphal density was observed. Also, the fungus showed an altered growth pattern when compared to the wild‐type growth, that is, when treated with the enzyme, hyper‐branching and tortuous hypha elongation resulting in a slower radial advance on the Petri dish was observed (Fig. S1).

## Discussion

In this study, we isolated a gene that codes for an endochitinase (ChiA Btt) of *B. thuringiensis* subsp. *tenebrionis* DSM‐2803*,* characterized its activity, and showed that the purified enzyme is active against *C. gloeosporioides,* an etiological agent of anthracnose in plants.

We cloned the chitinase gene and produced two versions of the enzyme in *E. coli*, ChiA Btt that harbors its putative native signal peptide, and recombinant 6x‐his tagged rChiA Btt that lacked its secretion signal peptide. ChiA Btt was found in the supernatant (i.e. protein is secreted), but not intracellularly (Fig. 1B), and rChiA Btt was located intracellularly but not in the supernatant (Fig. 3), this suggests that the ChiA Btt signal peptide is required for secretion of the enzyme in *E. coli*. Other studies have demonstrated that secretion signal peptide sequences in hydrolytic enzymes of *Bacillus* spp. or other bacteria are recognized by the secretion system of *E. coli* (Yamabhai et al. 2008; Casados‐Vázquez et al. 2015; Pelzer et al. 2015). Therefore, it is not surprising that ChiA Btt is secreted by *E. coli,* as observed in the present study. In addition, when the deduced sequence of ChiA Btt was compared with chitinases of *B. thuringiensis* and other bacterial species, its modular organization (catalytic domain, fibronectin‐like domain, and chitin‐binding domain) and signature residues in each module were highly conserved (Watanabe et al. 1993; Barboza‐Corona et al. 2003, 2008; Hardt and Laine 2004.; Tsuji et al. 2010; Arora et al. 2013). Perhaps, more unusual is the lower sequence conservation (~40% identity) in the second putative fibronectin‐like domain (ChiA Btt‐FLD2) in ChiA Btt when compared to other *B. thuringiensis* chitinases (Fig. 2). It is known that fibronectin generates extracellular and multifunctional matrices, which are important in cell bonding and may be implicated in substrate attachment, for example, binding/attachment to chitin (Jee et al. 2002). Also, chitin‐binding proteins are known to facilitate microbial attachment to chitin for subsequent degradation (Arora et al. 2013). Although we do not have any experimental support, it is possible that ChiA Btt‐FLD2 and a putative CBP of *B. thuringiensis* subsp. *tenebrionis* DSM‐2803 might act synergistically to attach to chitin for efficient hydrolysis of the substrate; a hypothesis to be confirmed in subsequent studies.

The occurrence of the smaller protein of 55 kDa is not surprising, and is most likely a processed derivative of the 74 kDa rChiA Btt. The processing of *B. thuringiensis* chitinases from the *C*‐terminal seems to be a common phenomenon (Thamthiankul et al. 2001; Casados‐Vázquez et al. 2015), and different evidence suggests that rChiA Btt is not an exception: (1) the rChiA Btt is 6x‐histidine tagged in the *N*‐terminal, which allows its purification using the Ni column, that is, if rChiA Btt was processed from the *N*‐terminal, we could not have co‐purified the ~55 kDa species with the ~74 kDa rChiA Btt. (2) Previously, using immunodetection with anti‐His antibody against rChiA74, we showed that this enzyme, an endochitinase with ~97% of identity with rChiA Btt, was processed from the *C*‐terminal (Casados‐Vázquez et al. 2015). Furthermore, the optimal temperature and pH ranges for rChiA were similar for other chitinases of *B. thuringiensis* but differed slightly (55°C, pH 6.5) when compared to activities of ChiA74 and ChiA HD73 obtained from crude extracts (Barboza‐Corona et al. 2003, 2008). Moreover, like other chitinases, rChiA Btt does not require a metal cofactor (Morales de la Vega et al. 2006; Casados‐Vázquez et al. 2015). In fact, all the metals tested here reduced ChiA Btt's activity, and only Hg^+2^ completely abolished enzymatic activity, probably because of interaction of Hg^+2^ with the S‐S or –SH group of rChiA Btt. A similar effect has been observed for rChiA74, and an exochitinase from *B. thurigiensis* subsp. *aizawai* (Morales de la Vega et al. 2006; Casados‐Vázquez et al. 2015). The Km of rChiA Btt is lower than the Km of other bacterial chitinases from *B. thuringiensis kenyae* (ChiA74, 2.15 *μ*mol L^−1^), *B. circulans* WL‐12 (ChiA1 Bc, 3.6 *μ*mol L^−1^), *S. marcescens* (BJL200‐ChiA, 4.2 *μ*mol L^−1^; BJL200‐ChiB, 6.8 *μ*mol L^−1^), and *Aeromonas caviae* (ChiA Ac, 129.3 *μ*mol L^−1^) (Watanabe et al. 1993; Lin et al. 1999; Synstad et al. 2008; Casados‐Vázquez et al. 2015), suggesting a higher affinity for the tetrameric fluorogenic substrate. In particular, the Km of rChiA Btt is 60% lower than the Km of rChiA74 using MU‐(GlcNAc)_3_ as a substrate (Casados‐Vázquez et al. 2015), although these proteins differ by only 10 amino acids. Further studies including site‐directed mutagenesis are required to determine the importance of these amino acid residues in lowering the Km of rChiA Btt when compared with rChiA74 (Takase 1993; Jee et al. 2002). Likewise, further studies are required to determine the influence of the ~55 kDa species in the kinetics of rChiA Btt.

Different studies have demonstrated that microbial chitinases are useful to control phytopathogenic fungi because of their ability to hydrolyze chitin in fungal cell walls (Asril et al. 2014). Previous studies have also shown that chitinases of *B. thuringiensis* and from other organisms inhibited phytopathogenic fungi, such as *Sclerotium rolfsii, Nigrospora sp, Aspergillus terreus, A. niger, Fusarium oxysporum,* and *F. moniliforme,* (Morales de la Vega et al. 2006; Awad et al. 2014; Suryanto et al. 2014; Karthik et al. 2015; Ni et al. 2015; Yu et al. 2015). However, crude extracts were used in most of these studies, and the effect may be due not only to the action of chitinases, but also due to other biomolecules. Here, we showed that a purified chitinase of a commercial strain of *B. thuringiensis* adversely affected the growth of *C. gloeosporioides*. To demonstrate this effect, two assays were performed, (1) by mixing the enzyme with the solid medium to test the effect on hyphal radial growth, and (2) using the well‐diffusion method. In the first assay, the effect correlated with the concentration of enzyme used, that is, the higher the rChiA Btt concentration the higher the level of inhibition of hyphal radial growth. In the well‐diffusion assays, we observed that purified chitinase produced hyper‐branching and tortuous hypha elongation of *C. gloeosporioides* resulting in a slower radial advance. This effect was probably due to faulty chitin microfiber crystallization induced by random chitinase activity, producing a defective cell wall assembly. In filamentous fungi, such as *Neurospora* crassa, growth proceeds by extension of hyphal tips and branching, but when fungus is grown under stress conditions, it is common to observe altered hyphal elongation accompanied by hyper‐branching that affects growth of the fungus (Gorovits and Yarden 2003).

In conclusion, our results suggest that ChiA Btt from *B. thuringiensis* subsp. *tenebrionis* DSM‐2803 could have applied value in controlling *C. gloeosporioides*.

## Conflict of Interest

No conflict of interest declared.

## Supporting information


**Figure S1.** Inhibition of rChiA Btt on the growth of *C. gloeosporioides* using the well diffussion assay. (A) The fungus was placed into the center of fresh potato dextrose agar and different chitinase concentrations were added in each well. Wells (a) without chitinase or added with (b) 18.75 U, (c) 9.38 U, (d) 4.69 U, (e) 3.34 U, and (f) 1.17 U. (B) Effect on the hyphal density and hyphae growth observed under light microscopy. Each small letter corresponds to the concentration using in (A).
