# Supplementary material for: The endochitinase ChiA Btt of Bacillus thuringiensis subsp. tenebrionis DSM‐2803 and its potential use to control the phytopathogen Colletotrichum gloeosporioides
Source: Microbiologyopen. 2016 May 12;5(5):819–29. doi: 10.1002/mbo3.372 (PMC5061718; doi:10.1002/mbo3.372)
Supplement: Supplementary file 1 — Figure S1. Inhibition of rChiA Btt on the growth of C. gloeosporioides using the well diffussion assay. (A) The fungus was placed into the center of fresh potato dextrose agar and different chitinase concentrations were added in each well. Wells (a) without chitinase or added with (b) 18.75 U, (c) 9.38 U, (d) 4.69 U, (e) 3.34 U, and (f) 1.17 U. (B) Effect on the hyphal density and hyphae growth observed under light microscopy. Each small letter corresponds to the concentration using in (A). [file MBO3-5-819-s001.pdf]

Supplementary Materials

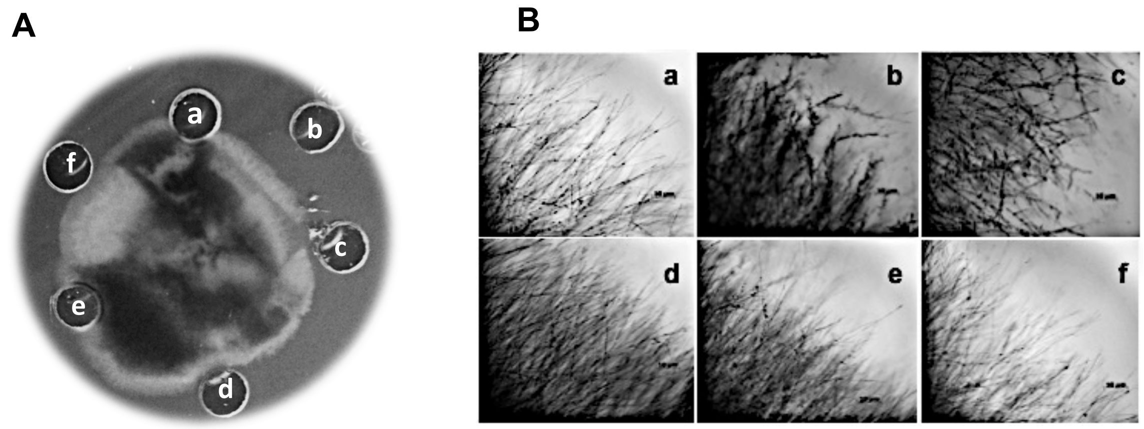

Fig. S1. Inhibition of rChiA Btt on the growth of *C. gloeosporioides* using the well-diffussion assay. (A) The fungus was placed into the center of fresh potato dextrose agar and different chitinase concentrations were added in each well. Wells (a) without chitinase or added with (b) 18.75 U, (c) 9.38 U, (d) 4.69 U, (e) 3.34 U, and (f) 1.17 U. (B) Effect on the hyphal density and hyphae growth observed under light microscopy. Each small letter corresponds to the concentration using in (A).
